# Artificial intelligence for genomic science: a scoping review of concepts, architectures, applications, and open challenges

**DOI:** 10.3389/fbinf.2026.1829576

**Published:** 2026-07-03

**Authors:** Wellington Francisco Rodrigues, Mariana T. D. Parise, Doglas Parise, Lucas Moraes dos Santos, Priscyla dos Santos Ribeiro, Paula Ristow, Mariana Santos Cardoso, Vasco Ariston de Carvalho Azevedo, Siomar de Castro Soares, Raquel Cardoso de Melo Minardi, Aristóteles Goés-Neto

**Affiliations:** 1 Molecular and Computational Biology of Fungi Laboratory, Department of Microbiology, Instituto de Ciências Biológicas, Universidade Federal de Minas Gerais, Belo Horizonte, Minas Gerais, Brazil; 2 Laboratory of Genetics, Biotechnology Institute, Federal University of Uberlândia, Uberlândia, Minas Gerais, Brazil; 3 Multidisciplinary Laboratory of Scientific Evidence, University Center of Mineiros (Unifimes), Mineiros, Goiás, Brazil; 4 Department of Computer Science, Universidade Federal de Minas Gerais, Belo Horizonte, Minas Gerais, Brazil; 5 Expertise Centre for Leptospirosis / WOAH Reference Laboratory for Leptospirosis, Department of Medical Microbiology and Infection Prevention, Amsterdam University Medical Centers, Amsterdam, Netherlands; 6 Laboratory of Cellular and Molecular Genetics, Institute of Biological Sciences, Universidade Federal de Minas Gerais, Belo Horizonte, Minas Gerais, Brazil; 7 Department of Microbiology, Immunology and Parasitology, Institute of Biological and Natural Sciences, Federal University of Triângulo Mineiro, Uberaba, Minas Gerais, Brazil

**Keywords:** artificial intelligence, deep learning, foundation models, genomics, large language models, machine learning, multi-omics

## Abstract

**Introduction:**

Artificial intelligence (AI) is becoming central to genomics and multi-omics, but its concepts, architectures, applications, evaluation standards, and translational requirements remain fragmented. This scoping review mapped how AI is defined and operationalized in genomic science, including machine learning, deep learning, graph-based methods, foundation models, and large language models, and synthesized their data modalities, applications, evaluation practices, interpretability strategies, and governance challenges.

**Methods:**

We conducted a PRISMA-ScR scoping review with Joanna Briggs Institute guidance. Eligible studies applied AI to genomics or closely allied omics in research, clinical, or public health contexts. MEDLINE/PubMed, Embase, and supplementary registers were searched from January 2001 to 3 September 2025 without language restrictions. Records were screened in duplicate, and standardized items were extracted, including AI concept or method family, omics modality, task, metrics, interpretability, governance, and deployment considerations. Methodological reporting and quality were appraised using design-appropriate JBI tools and summarized descriptively as a normalized 0%–100% checklist-fulfillment index.

**Results:**

From 3,785 records, 1,040 studies were included. Publication remained sparse until 2017 and then expanded steeply, with more than 90% appearing from 2018 onward. The normalized JBI checklist-fulfillment index was modest overall (mean 35.3%, SD 20.1; range 7.5%–87.5%) and was interpreted descriptively, not as a directly comparable quality score across designs. Conceptually, the field has moved from feature-engineered statistical learning toward representation learning systems modeling nucleotide sequences, regulatory context, single-cell states, multi-omics profiles, biomedical text, and clinical-genomic knowledge. Applications concentrated on variant interpretation, regulatory genomics, multi-omics integration, single-cell analysis, pathology/radiology-genomics fusion, and genomic decision support, with increasing use of deep learning, graph models, foundation models, and LLMs. Calibration, external validation, mechanistic interpretability, ancestry-aware fairness, privacy protection, and deployment models for sensitive genomic data were unevenly reported; prospective multisite evaluations were rare.

**Discussion:**

AI in genomics has scaled rapidly since 2017–2018, but translation remains constrained by heterogeneous concepts, inconsistent benchmarks, incomplete reporting, and limited governance. Priorities include biologically meaningful benchmarks; calibrated uncertainty for genomic decision support; mechanism-linked interpretability; ancestry- and site-aware validation; privacy-preserving analysis of sensitive genomic data; and human oversight for variant interpretation, precision medicine, and public health genomics.

**Systematic Review Registration:**

https://osf.io/uexzh.

## 1 Introduction

Artificial intelligence (AI) has become an increasingly important methodological layer in genomic science, supporting tasks that range from variant calling, regulatory annotation, and gene expression prediction to multi-omics integration, single-cell analysis, biomarker discovery, and clinical decision support. Earlier applications in genomics were largely built on feature-engineered statistical learning, including support vector machines, random forests, regularized regression, and probabilistic sequence models, which remain useful for structured genomic data, smaller cohorts, and interpretable clinical pipelines (Chen and Ishwaran, 2012; Dias and Torkamani, 2019; Huang et al., 2018; Okser et al., 2014). Over the last decade, however, the field has shifted toward deep representation learning, with convolutional, recurrent, attention-based, and transformer architectures learning regulatory motifs, variant effects, chromatin signals, and long-range sequence dependencies directly from biological data (Angermueller et al., 2016; Avsec et al., 2021; Sokolova et al., 2024; Zhou and Troyanskaya, 2015). This transition has expanded the analytical scope of genomics from task-specific prediction pipelines toward systems capable of integrating sequence, epigenomic, transcriptomic, proteomic, imaging, literature-based, and clinical information for discovery and translational inference (Baião et al., 2025; Ballard et al., 2024; Reel et al., 2021; Telenti et al., 2018).

More recently, foundation models and large language models (LLMs) have introduced a new conceptual and technical layer to genomic AI. DNA and RNA language models such as the Nucleotide Transformer and GENA-LM exemplify the use of large-scale pretraining to learn generalizable representations from genomic sequences (Dalla-Torre et al., 2024; Fishman et al., 2025). Single-cell and multi-omics foundation models, including scGPT and related large-scale architectures, extend this paradigm to cellular states and cross-modal biological representation learning (Bai et al., 2026; Cui et al., 2024; Rizvi et al., 2025). Tool-augmented and retrieval-augmented LLMs, such as GeneGPT and variant summarization systems grounded in curated databases, illustrate a complementary direction in which language models are connected to external biomedical resources to support genomic question answering, literature curation, variant interpretation, and evidence-grounded reporting (Du et al., 2026; Jin et al., 2024; Tang et al., 2024). These developments are promising, but they also introduce unresolved challenges related to factuality, calibration, hallucination, benchmark comparability, sensitive genomic data governance, and human oversight in high-stakes clinical or public health contexts (Bianchi et al., 2026; Coghlan et al., 2024; Rahimzadeh et al., 2022).

Several prior reviews have addressed important portions of this landscape. Reviews of deep learning in computational biology and genomics have clarified how neural networks can learn regulatory elements, noncoding variant effects, chromatin accessibility, and transcriptional regulation directly from sequence or epigenomic data (Angermueller et al., 2016; Barshai et al., 2020; Sokolova et al., 2024; Telenti et al., 2018). Other syntheses have focused on AI in cancer genomics, precision oncology, histopathology-genomics integration, and radiogenomics, highlighting applications in biomarker discovery, patient stratification, survival prediction, and molecularly guided treatment selection (Frasca et al., 2024; Gao et al., 2023; Unger and Kather, 2024; Xu et al., 2019). A further body of work has reviewed multi-omics integration, single-cell analysis, and machine learning across heterogeneous molecular modalities, emphasizing the value of deep generative models, graph-based methods, and multimodal fusion for complex biological systems (Baião et al., 2025; Ballard et al., 2024; Nicora et al., 2020; Reel et al., 2021). More recent papers have begun to synthesize LLMs, foundation models, code-generation benchmarks, and biomedical model evaluation, but these discussions often remain focused on specific model classes, datasets, or technical benchmarks rather than the full genomic research and translation ecosystem (Bianch et al., 2026; Dalla-Torre et al., 2024; Fishman et al., 2025; Tang et al., 2024; Wang et al., 2025).

Despite these contributions, the literature remains fragmented in ways that limit a panoramic understanding of AI in genomic science. First, prior reviews often focus on one methodological family, such as traditional machine learning, deep learning, graph models, or LLMs, rather than comparing how these families coexist across genomic tasks and evidence types. Second, many reviews are organized around a single data modality or application area, such as cancer, rare disease diagnosis, single-cell omics, imaging-genomics fusion, or variant interpretation, making it difficult to evaluate cross-cutting patterns in data modalities, model adaptation strategies, evaluation metrics, and deployment readiness (Dias and Torkamani, 2019; Prelaj et al., 2024; Roman-Naranjo et al., 2023; Unger and Kather, 2024). Third, key translational dimensions are unevenly integrated into existing syntheses, particularly probability calibration, external validation, ancestry and site bias, interpretability linked to biological mechanisms, privacy-preserving computation, protected health information handling, and governance for clinical implementation (Calvino et al., 2024; Fritzsche et al., 2023; Novakovsky et al., 2023; Rahimzadeh et al., 2022; Whalen et al., 2022). Consequently, it remains unclear whether the field is moving toward reproducible and clinically actionable genomic AI or whether many applications remain proof-of-concept systems with limited validation, comparability, and governance.

A scoping review is therefore appropriate because AI in genomics is methodologically broad, conceptually heterogeneous, and not suitable for a narrow meta-analysis of pooled effectiveness. The field includes classical machine learning, convolutional and recurrent neural networks, graph neural networks, transformer-based sequence models, foundation models, LLMs, retrieval-augmented systems, and tool-using agents. These methods are applied across DNA and RNA sequences, epigenomics, bulk and single-cell transcriptomics, proteomics, spatial assays, imaging-derived molecular signals, biomedical literature, and clinical narratives when these are used to support genomic inference. They are also evaluated with heterogeneous outcomes, including AUROC, AUPRC, F1 score, Matthews correlation coefficient, calibration metrics, expert review, factuality assessment, safety evaluation, and task-specific biological benchmarks (Avsec et al., 2021; Kan-Tor et al., 2024; Tang et al., 2024; Topçuoğlu et al., 2020). Mapping this diversity requires a design that can organize concepts, architectures, applications, evaluation practices, and governance issues without assuming that the included studies are directly comparable in design, population, task, or outcome.

The present review addresses this gap by systematically mapping the conceptual, methodological, and translational landscape of AI in genomics and closely allied omics domains. Specifically, we examine how AI methods are defined and operationalized across the literature; which model families and architectures are most frequently used; which genomic data modalities and application domains are prioritized; how performance, interpretability, calibration, and uncertainty are evaluated; and how governance issues, including privacy, fairness, protected health information, and deployment context, are reported. By integrating these dimensions, this review contributes a panoramic synthesis of AI in genomic science, with particular attention to the transition from classical machine learning and deep learning toward graph-based, transformer-based, foundation-model, and LLM-enabled systems. The aim is not to rank models or make pooled claims of comparative effectiveness, but to clarify how the field is structured, where evidence is strongest, and which methodological and governance gaps must be addressed before AI systems can be responsibly translated into genomic research, clinical genomics, precision medicine, and public health genomics.

## 2 Methods

### 2.1 Protocol and registration

This review was designed as a scoping review and conducted in accordance with the Preferred Reporting Items for Systematic Reviews and Meta-Analyses extension for Scoping Reviews (PRISMA-ScR) guidelines (Haddaway et al., 2022). The protocol was prospectively registered in the Open Science Framework (OSF; https://osf.io/uexzh) to ensure transparency and reproducibility. The review was guided by the following research question: How has artificial intelligence been applied to genomics and allied omics domains, and what are the main concepts, architectures, applications, evaluation practices, and open challenges reported in literature? Because the field of AI in genomics is methodologically broad, rapidly evolving, and heterogeneous in study design, we complemented the scoping review framework with descriptive quantitative mapping, bibliometric summaries, exploration topic modeling, and contextual methodological appraisal. These components were used to support evidence charting and interpretation, not to convert the review into a comparative effectiveness review or meta-analysis. Specifically, bibliometric analyses were used to describe temporal and geographic publication patterns; topic modeling was used as an exploratory tool to corroborate thematic patterns identified during charting; and critical appraisal was used to contextualize reporting and methodological variability across sources. No pooled effect estimates were calculated, and no included study was excluded based on methodological appraisal.

### 2.2 Eligibility criteria

We considered studies that explicitly applied artificial intelligence to genomics or closely allied omics domains across human and non-human systems and in research, clinical, public health, agricultural, or translational settings. For this review, “genomics” referred to analyses involving DNA or RNA sequence data, genome structure, gene regulation, genetic variation, genome annotation, gene or variant prioritization, genome-wide association, comparative genomics, functional genomics, or genome-informed decision support. “Closely allied omics” was operationally defined as omics modalities directly connected to genomic inference or genome-enabled interpretation, including epigenomics, transcriptomics, single-cell transcriptomics, spatial transcriptomics, proteomics, metagenomics, microbiome profiles, and multi-omics datasets in which genomic or transcriptomic information was a central component.

Eligible designs included original investigations, methodological and benchmarking papers, conference proceedings, reviews, scoping reviews, systematic reviews, perspectives, and preprints, provided that they contained substantive methodological, empirical, evaluative, or conceptual content relevant to AI in genomics. This broad inclusion was intentional and consistent with the aim of mapping a rapidly evolving field rather than estimating pooled intervention effects. To account for heterogeneity, studies were charted and synthesized according to study type, AI method family, omics modality, application domain, evaluation strategy, interpretability approach, and governance or deployment content. Reviews and perspectives were used primarily to map concepts, frameworks, challenges, and reported trends, whereas original, methodological, and benchmarking studies were used to characterize concrete models, datasets, tasks, and evaluation practices.

Text sources and clinical narratives were eligible only when they were explicitly used to support genomic inference, genomic decision support, variant interpretation, gene prioritization, literature-grounded genomic reporting, bioinformatics code generation, or integration of genomic findings with clinical or phenotypic information. For example, LLM studies using biomedical literature, clinical reports, phenotypic descriptions, or curated variant databases were included only when the task was directly linked to genomic or omics interpretation. Studies focused exclusively on general clinical NLP, radiology, pathology, or electronic health records without a genomic, transcriptomic, epigenomic, proteomic, metagenomic, or multi-omics linkage were excluded.

Large language and foundation models were included when they were trained on, evaluated with, or deployed for genomics-relevant tasks, including DNA/RNA/protein sequence modeling, variant interpretation, regulatory annotation, single-cell or multi-omics analysis, genomic question answering, literature curation for genomic evidence, or bioinformatics pipeline/code generation. We excluded studies whose AI applications were unrelated to genomics or allied omics, records lacking explicit omics content, and editorials or opinion pieces without methodological, empirical, evaluative, or conceptual substance. When both a preprint and a peer-reviewed version were available, the peer-reviewed article was retained. When only a preprint existed but provided sufficient methodological detail, including data, model, task, and evaluation description, it was eligible because of the rapid development cycle of AI and foundation-model research. Duplicate reports were consolidated, and inaccessible full texts were excluded after reasonable retrieval efforts.

### 2.3 Information sources

We searched MEDLINE/PubMed and Embase using database specific controlled vocabulary (MeSH/Emtree) and harmonized free text terms tailored to each platform. To capture gray literature and fast moving developments, we ran targeted searches in bioRxiv, medRxiv, and arXiv and screened conference abstracts and technical reports identified through society websites and institutional repositories. Reference lists of key reviews and benchmark papers were hand searched to maximize recall. No language restrictions were applied. We did not contact study authors to obtain additional data or pursue literature via direct outreach; all sources were obtained exclusively from bibliographic databases and public repositories.

All sources were searched from January 2001 through 03 September 2025. This time window (2001–2025) was selected because it encompasses the emergence, maturation, and consolidation of modern machine learning, deep learning, and genomics pipelines, capturing the full evolution of AI driven genomic analysis from early computational frameworks to contemporary foundation models. Full reproducible search strategies for each source, including exact queries and applied filters, are provided in Supplementary Material 1. Duplicates across databases and sources were removed before screening using automated matching followed by manual verification.

### 2.4 Search strategy

We combined controlled vocabulary, including MeSH and Emtree terms, with harmonized free-text terms and Boolean operators searched in titles and abstracts. No language restrictions were applied, and the prespecified search window covered January 2001 to 3 September 2025. The last search was executed on 3 September 2025, in accordance with PRISMA-ScR recommendations to report the most recent search date.

The search strategy was intentionally broad because the terminology used to describe AI in genomics has changed substantially over time. Earlier studies often used terms such as computational intelligence, machine intelligence, neural networks, computer reasoning, or knowledge representation, whereas more recent studies use terms such as machine learning, deep learning, transformer architectures, foundation models, and large language models. Similarly, genomic applications may be indexed under genomics, functional genomics, comparative genomics, structural genomics, transcriptomics, multi-omics, bioinformatics, or precision medicine. Therefore, we prioritized sensitivity during database searching to avoid missing older terminology, emerging model classes, or interdisciplinary studies that may not yet be consistently indexed.

To balance recall with specificity, broad retrieval was followed by duplicate removal, independent title/abstract screening, full-text eligibility assessment, and explicit exclusion of records that did not contain a genomics or closely allied omics component. During full-text review, studies were retained only when the AI method was directly linked to genomic or omics data, genomic inference, genome-informed clinical or public health decision support, variant interpretation, gene or pathway prioritization, or bioinformatics tasks. This staged approach allowed us to maximize capture of a rapidly evolving and inconsistently indexed field while removing records where AI was unrelated to genomic science.

Database-specific syntax, including explosion of subject headings, truncation, and adjacency operators where supported, was applied in MEDLINE/PubMed and Embase. Complete reproducible strategies for all sources are provided in Supplementary File 1. To ensure reproducibility, the complete PubMed strategy as executed is provided below. PubMed (MEDLINE) Search Strategy: (((((((Genomics [Title/Abstract]) OR (Comparative Genomics [Title/Abstract])) OR (Genomics, Comparative [Title/Abstract])) OR (Structural Genomics [Title/Abstract])) OR (Genomics, Structural [Title/Abstract])) OR (Functional Genomics [Title/Abstract])) OR (Genomics, Functional [Title/Abstract])) AND (((((((((((((((((((((Artificial Intelligence [Title/Abstract]) OR (Intelligence, Artificial [Title/Abstract])) OR (Computer Reasoning [Title/Abstract])) OR (Reasoning, Computer[Title/Abstract])) OR (AI [Title/Abstract])) OR (Machine Intelligence [Title/Abstract])) OR (Intelligence, Machine [Title/Abstract])) OR (Computational Intelligence [Title/Abstract])) OR (Intelligence, Computational [Title/Abstract])) OR (Computer Vision Systems [Title/Abstract])) OR (Computer Vision System [Title/Abstract])) OR (System, Computer Vision [Title/Abstract])) OR (Systems, Computer Vision [Title/Abstract])) OR (Vision System, Computer [Title/Abstract])) OR (Vision Systems, Computer [Title/Abstract])) OR (Knowledge Acquisition (Computer) [Title/Abstract])) OR (Acquisition, Knowledge (Computer) [Title/Abstract])) OR (Knowledge Representation (Computer) [Title/Abstract])) OR (Knowledge Representations (Computer) [Title/Abstract])) OR (Representation, Knowledge (Computer) [Title/Abstract])).

### 2.5 Selection of sources of evidence

**FIGURE 1:**
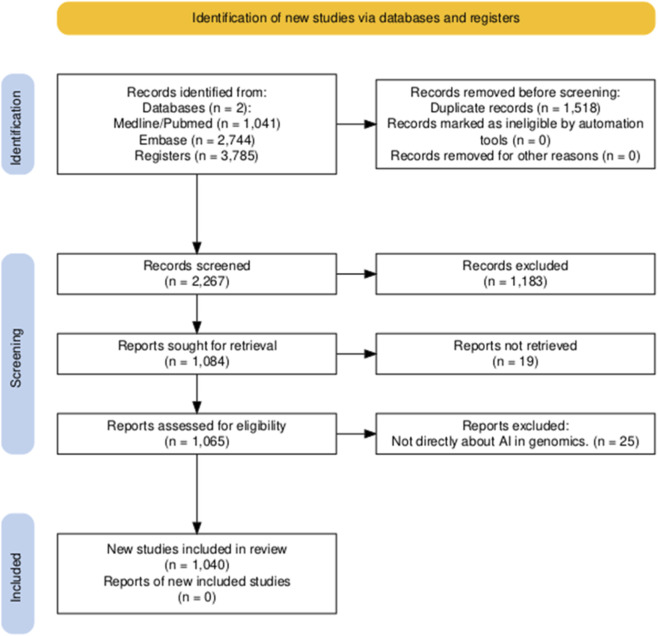
PRISMA 2020 flow diagram of study selection for AI in genomics. Records identified from MEDLINE/PubMed (n = 1,041) and Embase (n = 2,744), for a total of 3,785 records before deduplication. After removing 1,518 duplicates, 2,267 records were screened; 1,084 reports were sought for retrieval; 19 were not retrieved. Of 1,065 full text reports assessed for eligibility, 25 were excluded (not directly about AI in genomics). In total, 1,040 studies were included. Diagram generated with the PRISMA 2020 tool (Haddaway et al., 2022; Page et al., 2020).

All records were imported into Rayyan (Qatar Computing Research Institute) for systematic management. Duplicates were removed automatically and verified manually. Title and abstract screening were conducted independently by two reviewers (WFR and AGN), with disagreements resolved by a third reviewer. Eligible studies underwent full text review, also in duplicate, with reasons for exclusion documented. The process is summarized in a PRISMA 2020 flow diagram (Figure 1).

### 2.6 Data charting process

We extracted data using a structured form refined in a pilot on 20 studies to ensure consistent field definitions and coding rules. Data charting was performed in Covidence, which supported standardized form management, duplicate entry, discrepancy resolution, and controlled updates across reviewers. Two reviewers independently charted each included record, documented uncertainties, and reconciled discrepancies through discussion; a senior reviewer adjudicated any remaining disagreements.

The form captured bibliographic details, AI method family, genomic or omics modality, study type, application domain, evaluation metrics, interpretability approaches, governance and deployment considerations, and reported limitations. Multi label fields were allowed where appropriate; missing or unclear items were coded as not reported. Periodic internal audits were conducted to verify consistency and completeness before locking the dataset for analysis.

### 2.7 Data items

We charted a common set of variables for every included study to support consistent synthesis and comparison. For identification, we recorded authors, year of publication, country or region, and venue. Methodologically, we classified the primary AI approach into traditional machine learning, deep learning, graph-based models, or large language and other foundation models. We captured the genomic or omics modality addressed, including DNA or RNA sequence, epigenomics, bulk and single cell transcriptomics, proteomics, imaging derived signals, and text sources such as biomedical literature and clinical narratives when used to support genomic inference. We coded the application focus, spanning variant calling and interpretation, regulatory genomics, gene and variant prioritization, multi omics integration, clinical decision support, and program synthesis or code generation for bioinformatics tasks. Evaluation practices were abstracted as intrinsic metrics, such as accuracy, area under the ROC or precision recall curves, F1 score, Brier score, and probability calibration, and extrinsic assessments that included expert review, factuality checks, and safety or risk evaluations. We documented interpretability and governance elements, noting the use of attribution or explanation methods, explicit citation of evidence, handling of protected health information, de identification procedures, federated or on premises training, and references to regulatory frameworks. Finally, we recorded limitations and failure modes reported by the authors, including dataset or ancestry bias, hallucination or factual errors, reproducibility concerns, and computational or resource constraints. Multi label entries were permitted where applicable to reflect the heterogeneity of designs and outcomes.

### 2.8 Critical appraisal of individual sources of evidence

Critical appraisal is optional in scoping reviews; however, we included a structured methodological appraisal to contextualize the reporting quality and methodological variability of the included evidence. The purpose of this appraisal was descriptive and interpretive, not exclusionary. Appraisal results were not used to determine eligibility, to weight studies statistically, or to make direct claims about comparative effectiveness.

We used the Joanna Briggs Institute (JBI) Critical Appraisal Tools, selecting the checklist most appropriate to each study design. Two reviewers appraised each study independently and reconciled disagreements by consensus. Because the review included heterogeneous source types, including original studies, methodological papers, benchmarking studies, reviews, conference papers, perspectives, and preprints, we did not interpret JBI scores as directly interchangeable across designs. Instead, appraisal was used to identify broad patterns in reporting completeness, methodological transparency, validation practices, and potential limitations within each relevant evidence category.

For descriptive transparency, we summarized JBI appraisal results using a normalized percentage index from 0 to 100, calculated as the number of “Yes” items divided by the number of applicable items on the relevant checklist and multiplied by 100. Items marked “Not applicable” were excluded from the denominator, and items marked “Unclear” were treated as not meeting the criterion. This normalized index was used only as a pragmatic descriptive summary of checklist fulfillment within the constraints of each design-specific tool. It should not be interpreted as a universal quality score, as proof of methodological equivalence across study designs, or as a quantitative weight in the synthesis.

Where appraisal results are reported by strata, these strata are intended to contextualize the maturity and reporting completeness of the evidence base, not to re-rank studies or pool findings across designs. The full appraisal spreadsheet, including item-level decisions and applicable checklist domains for each study, is available in the public OSF repository linked to the protocol.

### 2.9 Synthesis of results

We synthesized the evidence using descriptive mapping, structured narrative synthesis, and exploration thematic analysis aligned with the scoping review objective. Given the heterogeneity of source types, model families, omics modalities, tasks, datasets, and evaluation metrics, no meta-analysis or pooled comparative effectiveness analysis was planned or conducted. Instead, synthesis was organized around five analytic lenses; concepts and definitions used to operationalize AI in genomics; method families and architectures, including traditional machine learning, deep learning, graph-based methods, foundation models, and LLMs; data modalities and application areas, including genomic sequence, epigenomics, bulk and single-cell transcriptomics, proteomics, metagenomics, spatial assays, imaging-derived molecular signals, and text used for genomic inference; evaluation, interpretability, and reliability practices, including discrimination metrics, calibration, external validation, attribution, explainability, factuality, and uncertainty; and governance and deployment considerations, including privacy, protected health information, de-identification, federated or on-premises training, fairness, and regulatory framing.

To account for heterogeneity, findings were charted and summarized by study type, AI method family, omics modality, application domain, and evidence function. Original investigations, methods papers, and benchmarking studies were used primarily to summarize implemented models, datasets, tasks, and evaluation practices. Reviews, perspectives, and conceptual papers were used primarily to map definitions, frameworks, emerging challenges, and translational considerations. Conference papers and preprints were interpreted cautiously and were used to capture emerging developments only when they reported sufficient methodological detail. This stratified synthesis prevented narrative claims from treating all source types as equivalent evidence.

Quantitatively, we conducted descriptive and bibliometric analyses to map temporal trends, geographic distribution, study type composition, and cumulative method adoption. These analyses were used to characterize the structure and evolution of literature rather than to infer causality or comparative effectiveness. Topic modeling of study objectives was used as an exploratory adjunct to support the thematic synthesis. Specifically, latent Dirichlet allocation was applied to identify recurring terms and topic patterns, which were then compared with manually charted themes. Topic modeling results were not treated as independent proof of thematic importance; rather, they served as a reproducibility-oriented check on whether machine-assisted text patterns were consistent with reviewer-coded themes.

All analytical workflows were implemented in R 4.3.2 (x86_64-w64-mingw32). Core packages included tidyverse 2.0.0 for data management, ggplot2 3.5.2 and ggrepel 0.9.6 for graphics, and the topicmodels package for latent Dirichlet allocation, with the exact version recorded in the repository sessionInfo output. Random seeds were fixed and reported in the analytical code. De-identified summary tables, CSV files, and figure scripts were exported for reproducibility, with exact queries and processing steps cross-referenced to Supplementary Material 1 and to the public OSF repository linked to the protocol.

## 3 Results

### 3.1 Scale of the literature and quality gradient across AI–genomics studies

A total of 3,785 records were identified across MEDLINE/PubMed and Embase. After removal of 1,518 duplicates, 2,267 records were screened by title and abstract, of which 1,183 were excluded. We sought 1,084 reports for full-text retrieval; 19 were unavailable. Among 1,065 full texts assessed for eligibility, 25 were excluded because they were not directly related to AI in genomics, resulting in 1,040 included studies (Figure 1).

Most exclusions occurred during title/abstract screening (1,183/2,267; 52.2%). The non-retrieval rate was low (19/1,084; 1.8%), and only 25 of 1,065 retrieved full texts were excluded at eligibility (2.3%). Methodological quality scores ranged from 7.5% to 87.5%, with a mean of 35.31% (SD 20.06%), corresponding to an overall low-to-moderate methodological quality profile with substantial dispersion. Most studies clustered in the low-to-intermediate range, whereas only a minority reached scores ≥70%. Per-study and domain-level methodological quality values are available in the OSF repository associated with this review.

### 3.2 Thematic distribution of AI methods, genomic modalities, and application domains

For each included study, we charted bibliographic information, study design, principal application area, AI method family, genomic or multiomic modality, target task, evaluation metrics, interpretability strategy, and governance or deployment considerations. Aggregated findings from these extracted variables are summarized below, while the complete per-study dataset is available in the Open Science Framework repository associated with the review protocol.

Across the included corpus, AI applications in genomics were distributed across five broad method families: classical machine learning, deep learning, graph-based models, foundation models, and LLM/RAG-based systems. Classical machine learning was most often associated with structured or feature-engineered data, including variant annotations, tabular omics, polygenic risk-related features, and clinical-genomic reporting tasks. Deep learning was mainly used in sequence-to-function modeling, regulatory variant prediction, protein representation learning, histopathology-genomics fusion, biomarker discovery, and multimodal prediction. Graph-based models were concentrated in molecular networks, regulatory connectivity, cell–cell relationships, and multiomic integration. Foundation models were increasingly represented in DNA, RNA, protein, methylome, single-cell, and multiomic applications. LLM/RAG-based systems appeared mainly in variant summarization, genomic question answering, clinical reporting, literature curation, and gene or variant prioritization.

The genomic and multiomic modalities most frequently represented included DNA and RNA sequences, variant data, gene expression matrices, epigenomic profiles, single-cell RNA/ATAC data, protein sequences, imaging-genomics data, and integrated multiomic datasets. Application domains were similarly diverse, including regulatory annotation, gene prediction, variant effect prediction, pathogenicity classification, cancer genomics, rare disease interpretation, single-cell annotation, biomarker discovery, risk prediction, clinical decision support, and biomedical knowledge extraction.

Evaluation strategies varied according to task. Classification and risk prediction studies commonly reported accuracy, AUROC, AUPRC, F1 score, Matthews correlation coefficient, sensitivity, specificity, and calibration metrics. Regulatory annotation and gene-prediction studies frequently used PR-AUC, MCC, and task-specific benchmark scores. Single-cell and multiomic integration studies commonly reported clustering and integration metrics, including normalized mutual information, adjusted Rand index, adjusted mutual information, silhouette, and batch-mixing scores. LLM/RAG-oriented studies used heterogeneous metrics, including accuracy, expert agreement, factuality assessment, retrieval quality, summarization metrics, and Pass@K for code-generation tasks.

When studies directly compared method families, reported performance advantages were task dependent. Deep learning and foundation models were more often reported as advantageous in studies involving long-range sequence context, sparse single-cell data, complex nonlinear interactions, or multimodal integration. However, classical machine learning remained competitive in smaller, tabular, cleaner, or more interpretable settings. Therefore, the evidence does not support a universal hierarchy of model superiority; rather, it indicates that performance depends on the interaction among data modality, sample size, task structure, validation design, and reporting quality. These patterns are summarized in Table 1, which provides a structured overview of method families, genomic modalities, application domains, evaluation metrics, and recurrent limitations across the included corpus.

**TABLE 1:** Summary of AI method families, genomic modalities, applications, evaluation patterns, and recurrent limitations across included studies.

Method family	Main genomic modality	Common applications	Typical metrics	Recurrent limitation
Classical ML	Tabular omics, variant features, PRS, clinical-genomic variables	Risk prediction, variant classification, expression classification, benchmarking baselines	AUROC, accuracy, F1-score, MCC, sensitivity/specificity	Dependence on feature engineering; limited modeling of complex nonlinear structure
Deep learning	DNA/RNA sequence, imaging, protein data, multiomics	Regulatory prediction, variant effect prediction, histopathology-genomics fusion, biomarker discovery	AUROC, AUPRC, F1-score, correlation, calibration metrics	Larger data requirements; interpretability and external validation challenges
Graph models	Molecular networks, regulatory graphs, cell–cell relationships	Multiomics integration, regulatory inference, biomarker discovery, pathway-level modeling	AUROC, clustering metrics, pathway-level validation, integration scores	Dependence on graph construction and prior biological knowledge
Foundation models	DNA, RNA, protein, methylome, single-cell, multiomics	Transfer learning, annotation, gene prediction, single-cell integration, regulatory modeling	MCC, AUPRC, NMI, ARI, task-specific benchmark scores	Compute demand, limited external validation, calibration gaps
LLM/RAG systems	Genomic text, variants, ClinVar/ClinGen, biomedical literature, EHR-linked data	Variant summarization, Q&A, clinical reporting, gene prioritization, code generation	Accuracy, expert agreement, factuality, retrieval metrics, Pass@K	Hallucination, retrieval coverage, governance, uncertainty quantification

AI, artificial intelligence; ML, machine learning; PRS, polygenic risk score; AUROC, area under the receiver operating characteristic curve; AUPRC, area under the precision-recall curve; F1-score, harmonic mean of precision and recall; MCC, matthews correlation coefficient; DNA, deoxyribonucleic acid; RNA, ribonucleic acid; LLM, large language model; RAG, retrieval-augmented generation; ClinVar, Clinical Variation database; ClinGen, Clinical Genome Resource; EHR, electronic health record; NMI, normalized mutual information; ARI, adjusted Rand index; Q&A, question answering; Pass@K, pass at k.

### 3.3 Bibliometric growth and geographic distribution of AI–genomics publications

A complementary bibliometric analysis was conducted using MEDLINE/PubMed, Embase, Web of Science, and SciELO to characterize temporal and geographic publication patterns in AI–genomics research from 2001 to August 2025. This analysis included 1,131 records. Annual output increased from 2 records in 2001 to 260 records during January–August 2025, corresponding to a 130-fold increase. Most records were published from 2018 onward (1,068/1,131; 94.4%).

**FIGURE 2:**
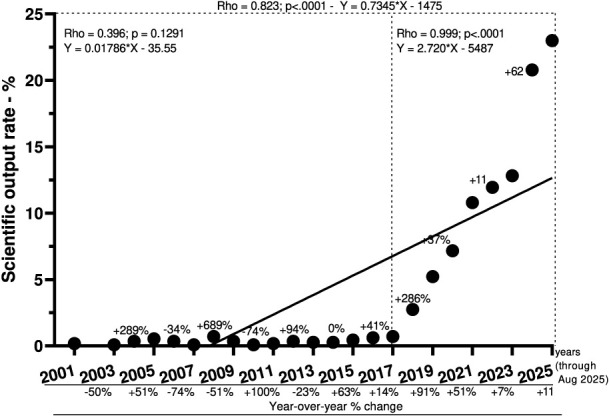
Temporal growth of AI–genomics publications, 2001–August 2025. Annual publication rates were estimated from MEDLINE/PubMed, Embase, Web of Science, and SciELO. The dashed vertical line marks the 2017–2018 inflection period identified by segmented trend analyses. Labels were restricted to selected reference years to improve readability. The 2025 estimate reflects January–August only.

Trend analyses supported a two-phase temporal pattern. Across the full period, annual publication rate was strongly correlated with calendar year (Spearman ρ = 0.823, 95% CI 0.621–0.923; p < 0.0001). Before 2017, the trend was not statistically significant (Spearman ρ = 0.396; p = 0.129; slope = 0.0179 percentage points/year). In contrast, the 2017–2025 period showed a near-monotonic increase (Spearman ρ = 1.000; p < 0.0001), with a steeper slope of 2.720 percentage points/year (95% CI 2.201–3.240; R^2^ = 0.956; p < 0.0001). These results identify 2017–2018 as the main inflection period in AI–genomics publication growth (Figure 2).

**FIGURE 3:**
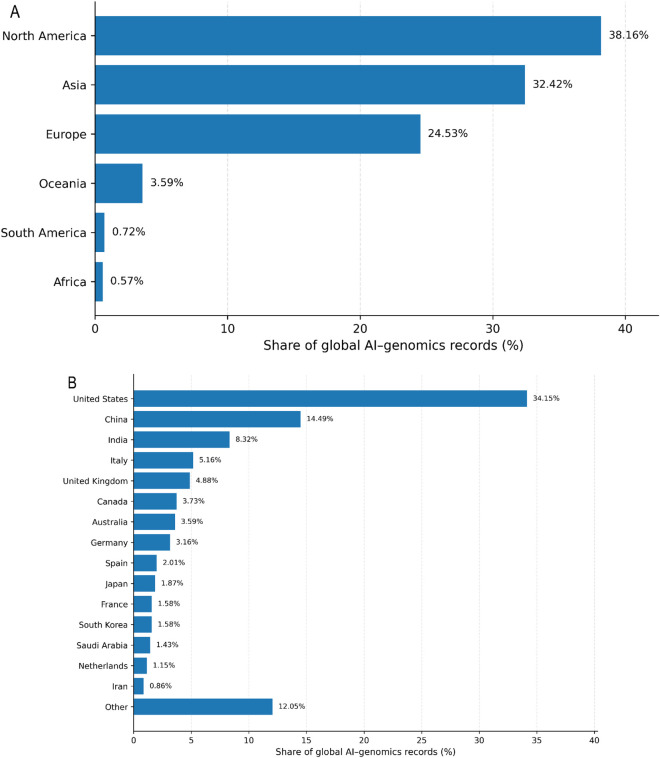
Geographic distribution of AI–genomics publications. **(A)** Continental distribution of records, showing concentration in North America, Asia, and Europe. **(B)** Top contributing countries, with countries outside the top 15 grouped as “Other”. Percentages represent shares of the global AI–genomics bibliometric record set. The 2025 estimate reflects January–August only. Percentages may not sum to exactly 100% because of rounding.

Geographically, AI–genomics publications were concentrated in three major regions (Figure 3A). North America accounted for the largest share of records (38.16%), followed by Asia (32.42%) and Europe (24.53%). Together, these three regions contributed approximately 95.12% of the global AI–genomics bibliometric record set. Smaller shares were observed for Oceania (3.59%), South America (0.72%), and Africa (0.57%), indicating a marked geographic imbalance in scientific production.

At the national level, the distribution was also highly concentrated and right-skewed (Figure 3B). The United States accounted for approximately one-third of all records (34.15%), followed by China (14.49%) and India (8.32%); together, these three countries represented approximately 56.96% of the total output. A second group of contributors included Italy, the United Kingdom, Canada, Australia, and Germany, each contributing between approximately 3% and 5% of the global record set. Additional countries contributed smaller shares, generally close to or below 2%, forming a long tail of lower-frequency national contributions. Overall, the top 10 countries accounted for approximately 80% of global output, and the top 20 for approximately 90%, indicating that AI–genomics publication activity was concentrated in a relatively small number of national producers.

Percentages represent relative shares of the global AI–genomics bibliometric record set and may not sum to exactly 100% because of rounding.

### 3.4 Temporal thematic patterns and text-mining findings

The thematic trajectory of the studies included three broad phases. First, studies published around 2015–2016 were mainly conceptual or infrastructural, focusing on EMR architecture, secure genomic data access, sequence-sharing infrastructure, and early ML applications in omics-scale biomarker discovery. Second, from 2017 to 2020, the literature shifted toward more concrete translational applications, including tumor sequencing interpretation, radiogenomics, regulatory genomics, variant calling, and early clinical decision-support systems. Third, from 2021 onward, the corpus increasingly emphasized multimodal integration, single-cell and spatial omics, graph-based learning, foundation models, explainability, federated learning, and governance. Representative high-volume or clinically oriented studies included AI-supported interpretation of 1,018 NG oncology cases, exome-based psychiatric genomics involving 5,090 samples, and pan-cancer multimodal prognostic modeling across 14 tumor types.

**FIGURE 4:**
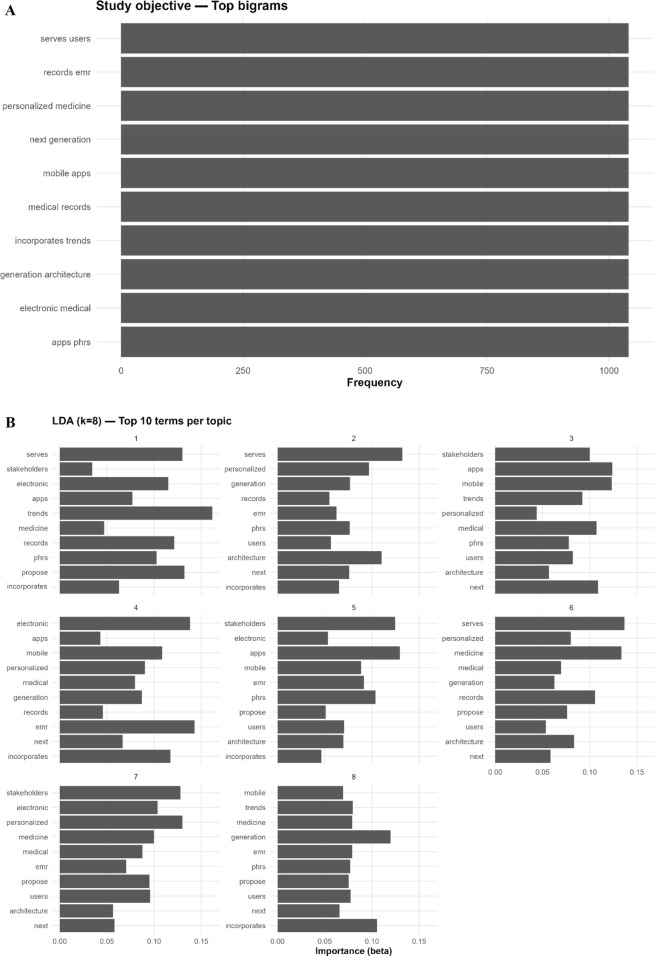
Text-mining and topic-modeling patterns extracted from study objectives. **(A)** Most frequent bigrams identified after tokenization and Portuguese/English stop-word and domain-filler removal. The leading bigrams were mainly related to next-generation EMR architecture, mobile applications, personal health records, stakeholder-oriented systems, and personalized medicine. **(B)** LDA topic modeling of study objectives using k = 8 topics. Top terms by β weight were grouped into four recurring motifs: EMR modernization, mobile/PHR ecosystem, architecture and stakeholders, and personalized medicine. LDA was trained on study objectives using terms appearing in at least 10 documents; β represents term weight within each topic and γ represents topic proportion per document.

Text mining of study objectives identified high-frequency bigrams related to EMR infrastructure, mobile/PHR ecosystems, stakeholder-oriented architecture, and personalized medicine. The most frequent bigrams included “next-generation,” “generation architecture,” “electronic medical,” “medical records,” “records EMR,” “mobile apps,” “apps PHRs,” “serves users,” “incorporates trends,” and “personalized medicine” (Figure 4A).

LDA topic modeling with k = 8 yielded topics that could be grouped into four recurring motifs: EMR modernization, mobile/PHR data capture, architecture and stakeholders, and personalized medicine. The leading terms across these topics included “emr,” “electronic,” “records,” and “incorporates” for EMR modernization; “apps,” “mobile,” “phrs,” and “users” for mobile/PHR ecosystems; “architecture,” “serves,” “stakeholders,” and “propose” for system design; and “personalized,” “medicine,” “trends,” and “next/generation” for personalized medicine (Figure 4B).

Together, these text-mining patterns indicate that a subset of the corpus framed AI–genomics integration through health informatics infrastructure, data capture systems, and personalized medicine architectures, rather than through model development alone.

### 3.5 Historical distribution of AI method milestones in genomics

A milestone-based synthesis of 41 peer-reviewed records published between 2004 and 2024 showed a temporal shift in the dominant AI approaches used in genomics and closely related omics domains. Earlier milestones were mainly represented by traditional machine learning or other/unspecified statistical learning approaches, including HMMs, support vector machines, random forests, and related probabilistic or feature-engineered methods. From the mid-2010s onward, deep learning milestones accumulated more rapidly, particularly in regulatory genomics, variant calling, proteomics, computational pathology, and multimodal prediction.

**FIGURE 5:**
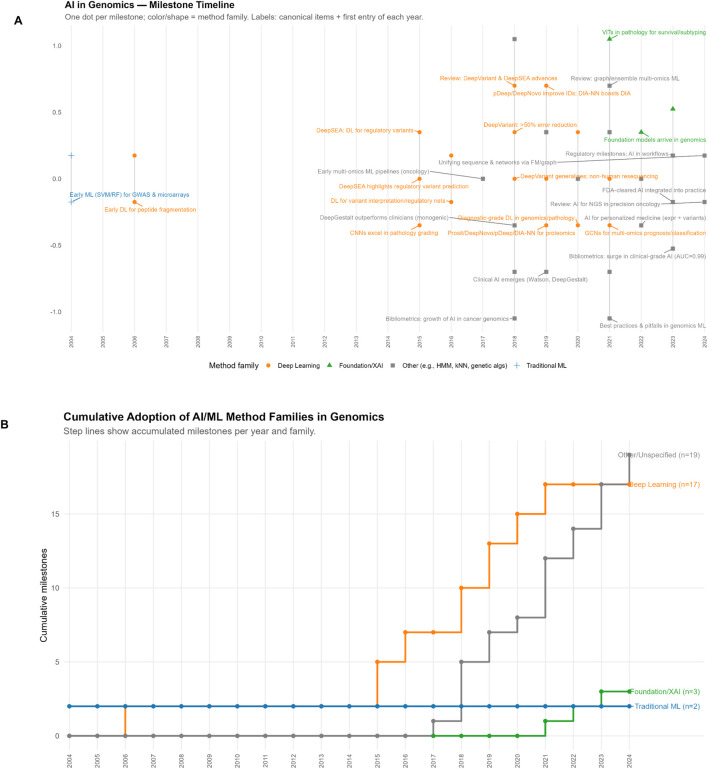
Milestones and cumulative adoption of AI in genomics (2004–2024). **(A)** Milestone timeline. Each dot marks a peer-reviewed milestone (n = 41) positioned by year; the vertical jitter has no quantitative meaning and is used only to avoid overlap. Color/shape encode the method family: Traditional ML, Deep Learning, Foundation/XAI, and Other/Unspecified (e.g., HMM/kNN/unspecified or mixed ML). Canonical items are labeled, including early deep nets for proteomics (2006), DeepSEA for regulatory variant prediction (2015), DeepVariant for germline calling (2018), weakly supervised WSI cancer detection (2019), multimodal histology–genomics fusion (PathomicFusion, 2021), Vision Transformers in pathology (2021), and the emergence of foundation models in genomics (2022), alongside reports of FDA cleared AI tools entering clinical workflows (2023). **(B)** Cumulative counts by method family. Step curves show the accumulation of milestones per year. By 2024 the totals are: Other/Unspecified (n = 19), Deep Learning (n = 17), Foundation/XAI (n = 3), and Traditional ML (n = 2). The trajectory highlights a mid-2010s inflection driven by deep learning, followed by early but growing activity around foundation models and explainability/federated approaches. Abbreviations: CNN, convolutional neural network; ViT, Vision Transformer; WSI, whole slide imaging; FM, foundation model; XAI, explainable AI; NGS, next-generation sequencing.

Figure 5A positions selected peer-reviewed milestones by year and highlights representative methodological transitions, including early neural-network applications in proteomics, DeepSEA for regulatory variant prediction, DeepVariant for germline variant calling, weakly supervised whole-slide imaging models, multimodal histology–genomics fusion, Vision Transformers in pathology, and emerging foundation-model applications in genomics. Figure 5B summarizes cumulative adoption by method family through 2024. Cumulative milestone counts were highest for Other/Unspecified methods (n = 19) and Deep Learning (n = 17), followed by Foundation/XAI approaches (n = 3) and Traditional ML (n = 2).

Overall, this milestone mapping indicates a shift from earlier feature-engineered and probabilistic approaches toward deep learning, multimodal modeling, and emerging foundation/XAI-oriented methods. These patterns should be interpreted as a descriptive synthesis of selected methodological milestones rather than as evidence of universal superiority of any model family across genomic tasks (Figures 5A,B).

### 3.6 Method-family-specific patterns: traditional machine learning

Traditional machine learning studies were mainly associated with structured, feature-engineered genomic and omics datasets, including variant annotations, gene expression profiles, GWAS/PRS-derived variables, microbiome features, epigenomic markers, regulatory annotations, and clinical-genomic variables. The most frequently reported algorithmic families included support vector machines, random forests, gradient boosting frameworks, regularized regression models, decision trees, k-nearest neighbors, Naive Bayes, hidden Markov models, and Bayesian networks. Across the corpus, these approaches were generally used when predictors could be represented as tabular, sequence-derived, pathway-derived, or biologically annotated features.

The main application domains included phenotype and clinical risk prediction, variant pathogenicity and functional-effect classification, GWAS/PRS-related modeling, gene expression-based classification, somatic mutation calling, microbiome and 16 S rRNA-based classification, epigenomic state classification, regulatory modeling, and rare-disease diagnostic workflows. Traditional ML was also frequently used as a benchmarking strategy against more complex architectures, particularly in studies involving modest sample sizes, structured omics data, or settings in which feature-level interpretability was important.

Reported evaluation practices most often included AUROC, AUPRC, accuracy, sensitivity, specificity, F1-score, Matthews correlation coefficient, R^2^, RMSE, MAE, and, less frequently, calibration metrics. Cross-validation, out-of-bag estimation for random forests, and external or held-out validation were reported in studies with more rigorous evaluation designs. Recurrent methodological considerations included feature engineering, dimensionality reduction, feature selection, normalization, missing-data handling, class-imbalance correction, ancestry or population-structure adjustment, and prevention of data leakage.

Across the studies included, traditional ML was most often positioned as a competitive baseline or preferred approach for smaller, tabular, high-dimensional, or feature-engineered genomic datasets. Its recurrent advantages were interpretability, data efficiency, and compatibility with biologically meaningful predictors, whereas recurrent limitations included dependence on feature engineering, sensitivity to preprocessing choices, limited capacity to model complex nonlinear or long-range biological structure, and reduced generalizability when validation was restricted or population structure was insufficiently addressed.

### 3.7 Method-family-specific patterns: deep learning

Deep learning studies were concentrated in sequence-to-function modeling, regulatory variant prediction, chromatin accessibility, transcription factor binding, histone-mark prediction, gene expression prediction, splicing-related tasks, methylation detection, variant calling, single-cell analysis, multimodal integration, histopathology–genomics fusion, and other genomic signal-annotation tasks. The main data modalities included raw DNA/RNA sequence, one-hot nucleotide matrices, k-mer or learned embeddings, NGS and ONT read-derived representations, pileup images, epigenomic tracks, gene expression matrices, single-cell omics, and multimodal combinations of sequence, chromatin, expression, imaging, and clinical data.

CNN-based architecture was frequently reported for motif discovery, local sequence features, chromatin accessibility modeling, transcription factor binding prediction, and regulatory variant interpretation. Recurrent architecture, including LSTM- and GRU-based models, were used to capture broader contextual dependencies across genomic windows. Attention-based and Transformer-derived models were increasingly associated with long-range regulatory interactions, enhancer–promoter inference, gene expression prediction from extended genomic contexts, and multi-task outputs. Autoencoders and variational autoencoders were mainly represented in unsupervised representation learning, denoising, dimensionality reduction, and single-cell or multiomic integration, whereas graph-aware variants were used to model cellular or molecular relationships.

Across the corpus, representative deep learning systems included DeepSEA, Basset, ExPecto, Enformer, and DeepVariant, reflecting recurrent applications in sequence-to-chromatin prediction, variant-effect estimation, long-range regulatory modeling, and variant calling. Training practices reported across studies included one-hot or binary encoding of DNA sequences, k-mer embeddings, pileup-image representations for variant calling, reverse-complement augmentation or shared-weight designs, dropout, early stopping, weight decay, class weighting, undersampling, synthetic examples, and augmentation strategies for noisy genomic labels. Several studies also emphasized train/validation/test splitting strategies designed to avoid coordinate leakage or overoptimistic performance estimates.

Evaluation most often relied on AUROC, AUPRC, accuracy, F1-score, correlation metrics for continuous genomic signals, chromosome-held-out validation, k-fold validation, platform-specific benchmarking for variant calling, and, less frequently, calibration assessment. Interpretability strategies included *in silico* mutagenesis, Integrated Gradients, DeepLIFT, saliency or CAM-based approaches, SHAP, motif extraction from convolutional filters, and inspection of attention maps. Recurrent limitations included larger data requirements, sensitivity to class imbalance and noisy labels, risk of data leakage, limited external validation, limited calibration reporting, and variability in the biological validation of model-derived explanations.

### 3.8 Method-family-specific patterns: foundation models and LLM/RAG systems

Foundation-model and LLM/RAG studies were concentrated in DNA/RNA sequence modeling, epigenomic prediction, single-cell annotation, variant summarization, genomic question answering, clinical reporting, literature curation, gene and variant prioritization, perturbation reasoning, pathway inference, drug sensitivity prediction, and automated code generation for bioinformatics pipelines. The models reported across this subset included general-purpose LLMs such as GPT-4/4o, Llama variants, and Qwen, as well as domain-specific genomic models, including DNABERT-2, Nucleotide Transformer, GENA-LM, Omni-DNA, EpiGePT, GeneGPT, and Geneverse. These systems were applied to both discriminative and generative tasks, using DNA and RNA sequences, protein sequences, single-cell transcriptomics, epigenomic tracks, structured variant resources, biomedical literature, EHR-linked clinical text, spatial transcriptomics, and imaging-related data.

Architecturally, Transformer-based models predominated, including encoder, decoder, and encoder–decoder designs. Long-context and domain-adapted architectures were used for extended genomic sequences and regulatory modeling, while tool-augmented and retrieval-augmented systems were used for tasks requiring access to external genomic or clinical knowledge bases. Adaptation strategies included domain-specific pretraining, supervised fine-tuning, instruction tuning, reinforcement learning from human feedback, LoRA/PEFT-based parameter-efficient tuning, retrieval-augmented generation over curated resources such as ClinVar and ClinGen, and API-based tool integration. In the included studies, RAG and tool-augmented approaches were primarily used to improve factual grounding, provenance, and access to curated genomic evidence.

Reported performance varied substantially by task, benchmark, and evaluation design. Selected studies reported 17% top-50 accuracy for GPT-4 in rare-disease gene prioritization, average MCC of 0.767 for Omni-DNA across 18 of 26 benchmark tasks, F1 ≈ 93.7 for GENA-LM in promoter prediction, PCC ≈0.787 for EpiGePT in cross-cell-type epigenomic prediction, recall of 97.85% for fine-tuned models in causative variant identification, near-perfect expert agreement for RAG-grounded variant summaries, and Pass@20 ≈ 50% for GPT-4 in code-generation tasks. These values indicate strong performance in selected settings, but they do not establish uniform superiority across genomic tasks because benchmarks, datasets, input modalities, and validation designs differ widely.

Evaluation metrics included accuracy, F1-score, MCC, Pearson correlation, AUROC, AUPRC, BLEU/ROUGE, BERTScore, distributional distances for single-cell annotation, Pass@K for code generation, retrieval quality measures, factuality checks, expert review, and safety scoring. Benchmarking efforts included Genomic Touchstone for DNA/RNA/protein modeling, BioCoder for code generation, CARDBiomedBench for biomedical Q&A and safety, and specialized datasets for variant summarization and enhancer prediction. However, heterogeneity in preprocessing, benchmark design, task definitions, and reporting practices limited direct comparability across studies.

Interpretability and grounding strategies were mainly based on evidence citation, attribution to retrieved sources, RAG pipelines, saliency mapping for sequence models, motif-level relevance analysis, and API-based tool use for traceability. Recurrent limitations included hallucination, incomplete retrieval coverage, overclassification bias, dataset and domain shifts, inconsistent safety and fairness evaluation, limited calibration, limited uncertainty quantification, compute demands, reproducibility gaps, and incomplete biomedical knowledge coverage. Zero-shot performance was reported as insufficient for several structured omics or clinical genomics tasks, with fine-tuning, retrieval augmentation, or human expert review often required for more reliable outputs.

Governance and deployment considerations were unevenly reported. Although many genomic LLMs and foundation models were released through open repositories or model hubs, explicit discussion of protected health information, de-identification, federated training, on-premises deployment, clinical accountability, and human-in-the-loop oversight was limited. Overall, the reviewed studies show rapid expansion of foundation-model and LLM/RAG approaches in genomics, but also highlight persistent gaps in standardized evaluation, calibration, interpretability, factual grounding, governance, and clinical validation.

## 4 Discussion

This scoping review mapped 1,040 studies on artificial intelligence (AI) in genomics and multiomics, covering classical machine learning, deep learning, graph-based models, foundation models, and LLM/RAG-based systems. The revised Results show that the field is broad, rapidly expanding, and methodologically heterogeneous. Three findings are particularly important for interpreting the literature. First, the evidence base is large but uneven in methodological quality, with scores ranging from 7.5% to 87.5% and a mean of 35.31% (SD 20.06%), indicating an overall low-to-moderate quality profile. Second, AI applications are distributed across distinct method families, genomic modalities, application domains, and evaluation practices, rather than forming a single homogeneous field. Third, bibliometric and thematic analyses indicate that AI–genomics research accelerated sharply after 2017–2018 and increasingly shifted toward multimodal integration, single-cell and spatial omics, foundation models, explainability, and governance. Together, these findings directly address the aims of this review: to map the main AI method families, characterize their genomic and multiomic applications, and examine how evaluation, interpretability, translation, and governance have been addressed across the literature.

A central implication of these results is that AI methods in genomics should be interpreted according to task, data modality, sample size, validation design, and reporting quality. The evidence does not support a universal hierarchy in which one model family is consistently superior across genomic tasks. Instead, the extracted patterns indicate a division of labor among method families. Classical ML was most frequently associated with structured or feature-engineered variables, including tabular omics, variant annotations, polygenic risk score-related features, microbiome profiles, epigenomic markers, and clinical-genomic variables. This pattern is consistent with studies showing that support vector machines, random forests, regularized regression, and gradient-boosting models remain useful in high-dimensional but structured genomic settings, particularly when interpretability, smaller sample sizes, and feature-level explanations are important (Chen and Ishwaran, 2012; Goldstein et al., 2011; Okser, Pahikkala, Airola, Salakoski, Ripatti and Aittokallio, 2014).

In contrast, deep learning and foundation models were more often reported as advantageous in settings involving long-range sequence context, nonlinear interactions, sparse single-cell data, or multimodal integration. DeepSEA and related sequence-to-function models illustrate the use of deep neural networks for regulatory prediction and variant-effect estimation from DNA sequence features (Zhou and Troyanskaya, 2015). DeepVariant illustrates the use of deep learning in germline variant calling, with reported error reductions exceeding 50% compared with earlier approaches in the original benchmark context (Poplin et al., 2018). Enformer further exemplifies the move toward long-range regulatory modeling by using extended sequence context for gene expression and chromatin-related prediction tasks (Avsec et al., 2021). These examples support a more specific interpretation: representation learning is particularly useful when biological signals depend on complex sequence structure, distal regulatory relationships, or multimodal input. They should not be interpreted as evidence that deep learning or foundation models are universally preferable.

The same task-dependent interpretation applies to foundation models and LLM/RAG systems. The Results showed strong performance in selected benchmark settings, including MCC = 0.767 for Omni-DNA across 18 of 26 tasks, F1 ≈ 93.7 for GENA-LM in promoter prediction, PCC ≈0.787 for EpiGePT in cross-cell-type epigenomic prediction, and Pass@20 ≈ 50% for GPT-4 in code-generation tasks. These findings demonstrate the promise of large pretrained models in specific tasks, but they also highlight the heterogeneity of benchmarks, input modalities, and validation strategies. Therefore, the main implication is not that foundation models replace classical ML or deep learning, but that they expand the methodological repertoire for transfer learning, sequence modeling, variant summarization, single-cell annotation, biomedical question answering, and retrieval-grounded genomic reporting (Dalla-Torre et al., 2024; Fishman et al., 2025; Tangm et al., 2024).

The application landscape identified in the Results was also structured rather than diffuse. AI–genomics applications were concentrated in recurrent domains, including regulatory annotation, variant calling and interpretation, cancer genomics, biomarker discovery, risk prediction, single-cell annotation, multiomic integration, clinical reporting, and biomedical knowledge extraction. This concentration suggests that AI is being repeatedly applied to areas where genomic data are high dimensional, heterogeneous, and difficult to interpret using conventional analytical pipelines. Cancer genomics and precision oncology remain especially prominent. AI-supported interpretation of 1,018 NG oncology cases illustrates the role of AI in clinical variant interpretation and trial matching (Patel et al., 2018). Pan-cancer multimodal prognostic modeling across 14 tumor types further shows how histology and genomics can be integrated for risk prediction (Chen et al., 2022). Radiogenomics and computational pathology extend this translational thread by connecting molecular profiles with imaging and tissue-level phenotypes, although much of the evidence remains methodological or retrospective rather than prospective and multisite (Unger and Kather, 2024).

Variant interpretation and genotype–phenotype mapping also emerged as central clinical genomics use cases. Benchmarking and review studies emphasize variant calling, curation concordance, explainability, and natural language processing for genomic interpretation (Abdelwahab et al., 2023; Dias and Torkamani, 2019; Telenti et al., 2018; Zhou et al., 2019). The inclusion of LLM/RAG systems expands this domain by introducing variant summarization, genomic question answering, and guideline-oriented reporting. However, these uses also intensify the need for factual grounding, uncertainty reporting, and traceability, because errors in variant interpretation may have direct clinical consequences. Beyond oncology and variant interpretation, the review identified applications in rare diseases, pharmacogenomics, immune oncology, infectious diseases, cardiovascular genomics, public health genomics, agriculture, and One Health. Examples include transcriptomic prediction in acute myeloid leukemia (Warnat-Herresthal et al., 2020), AI-assisted SARS-CoV-2 diagnostics (Rohaim et al., 2020), ML-supported exome prioritization in neurodevelopmental disorders (Dingemans et al., 2022), and genomic approaches related to climate-resilient breeding and plant or animal health (Harfouche et al., 2019; Thudi et al., 2021). These examples show that the scope of AI in genomics extends beyond human precision medicine, even though the strongest concentration of studies remains in biomedical and oncology-oriented applications.

One finding is that part of the AI–genomics literature is concerned not only with model development, but also with the information systems required to make AI-enabled genomics usable. The text-mining analysis identified high-frequency bigrams related to “electronic medical,” “medical records,” “records EMR,” “mobile apps,” “apps PHRs,” “serves users,” and “personalized medicine”. The LDA analysis similarly grouped study objectives into motifs related to EMR modernization, mobile/PHR data capture, architecture and stakeholders, and personalized medicine. These findings suggest that health informatics infrastructure represents an important translational layer for AI-enabled genomics. Genomic prediction alone is insufficient for clinical implementation; genomic and phenotypic information must be captured, harmonized, interpreted, and returned within usable clinical or public health workflows.

In this context, EMR-centered architectures, PHRs, mobile data capture, and stakeholder-oriented systems may function as integration points where genomic interpretation, risk stratification, decision support, and clinical reporting can be connected. However, the Results also show that governance and deployment considerations remain unevenly reported, particularly for foundation models and LLM/RAG systems. This creates a gap between model capability and implementation readiness. The implication is that future translation of AI-enabled genomics will depend not only on higher AUROC, AUPRC, MCC, or F1-score, but also on interoperability, provenance, privacy protection, auditability, and clinical usability. Legal and ethical analyses similarly emphasize consent, data-use governance, group risk, and GDPR-aligned data sharing in genomic AI contexts (Chapman et al., 2025; Rahimzadeh et al., 2022; Townend, 2018). Studies on privacy-preserving analytics, federated learning, and blockchain-enabled infrastructures further indicate possible strategies for managing sensitive genomic data across institutions (Calvino et al., 2024; Kuo et al., 2020).

The most consistent translational bottlenecks identified across the review were limited external validation, heterogeneous benchmarks, incomplete calibration reporting, uneven interpretability, and underdeveloped governance. These limitations appeared across method families, but they were especially consequential for deep learning, graph-based models, foundation models, and LLM/RAG systems. The Results showed that evaluation strategies varied widely by task, including AUROC, AUPRC, F1-score, MCC, clustering metrics, integration scores, factuality assessment, retrieval metrics, expert agreement, and Pass@K. This diversity is appropriate given the range of tasks, but it also limits direct comparability across studies. For clinical or translational applications, internal discrimination metrics are not enough. Models used for risk prediction, variant interpretation, or clinical reporting also require calibration, external validation, and decision-relevant endpoints. Several studies reported cross-validation or held-out validation, but the broader evidence base remains weighted toward retrospective evaluations, benchmarking studies, and methodological demonstrations. This pattern explains why the review can map promising applications but should not be interpreted as establishing comparative clinical effectiveness across model families.

Interpretability remains similarly uneven. Classical ML offers feature-level interpretability through coefficients, tree importance, or simpler model structures, whereas deep learning and foundation models often require post-hoc methods such as saliency maps, *in silico* mutagenesis, DeepLIFT, Integrated Gradients, SHAP, attention inspection, or motif extraction. These methods can help connect model behavior to biological signals, but their clinical meaning is not always validated. In this respect, interpretability should not be treated as a visual add-on; it should be evaluated against biological plausibility, known regulatory mechanisms, experimental evidence, and clinical usability requirements. In silico mutagenesis and attribution-based methods have been used to interrogate sequence models and regulatory predictions (Avsec et al., 2021; Shrikumar et al., 2017), while interpretable architectures and visualization approaches have been proposed to improve biological insight from genomic models (Novakovsky et al., 2023). Nevertheless, the literature still lacks consistent criteria for determining when an explanation is sufficiently reliable for clinical or translational decision-making.

Governance is the least mature dimension of the literature. The Results showed that LLM/RAG and foundation-model studies frequently discussed hallucination, retrieval coverage, uncertainty quantification, reproducibility, and safety, but explicit treatment of protected health information, de-identification, federated training, on-premises deployment, clinical accountability, and human-in-the-loop oversight was limited. For LLM/RAG systems, this is especially important because factuality and traceability depend not only on model architecture but also on retrieval quality, database coverage, prompt design, and evidence attribution. RAG and tool-augmented systems may improve provenance and access to curated genomic evidence, but they do not eliminate the need for expert review, uncertainty communication, and governance standards.

The complementary bibliometric analysis provides additional context for these methodological and translational patterns. AI–genomics publication output increased sharply after 2017–2018: annual output rose from 2 records in 2001 to 260 records during January–August 2025, and 1,068 of 1,131 bibliometric records were published from 2018 onward. This acceleration helps contextualize the methodological diversity observed in the scoping review, because the field expanded during the same period in which deep learning, multimodal integration, single-cell analysis, and foundation models became more prominent. However, this bibliometric trend should be interpreted as evidence of publication growth, not as proof of clinical maturity.

The geographic analysis also has important implications. The Results showed that AI–genomics publications were concentrated in North America, Asia, and Europe, with these three regions accounting for approximately 95% of the bibliometric record set. At the country level, the United States, China, and India accounted for more than half of the total output. This concentration suggests that the global evidence base may be shaped disproportionately by a limited set of national research ecosystems. Such asymmetry matters because genomic datasets, health infrastructures, ancestry composition, computing resources, and regulatory environments differ across regions. As a result, models trained and validated primarily in high-output countries may not generalize equally well to underrepresented populations or lower-resource health systems. This geographic imbalance also intersects with the governance and fairness concerns identified in the thematic synthesis. If AI–genomics tools are developed primarily in settings with greater computational resources, richer genomic databases, and stronger research infrastructure, then their deployment in less represented regions may reproduce or amplify existing inequities. Addressing this issue will require more inclusive datasets, transparent reporting of ancestry and site composition, regionally diverse validation cohorts, and collaborative governance models that include researchers and health systems from underrepresented regions.

The nature of the evidence base and the scoping approach entail several limitations. Although the search strategy covered MEDLINE/PubMed, Embase, Web of Science, and SciELO, and was supplemented by targeted searches in bioRxiv, medRxiv, and arXiv, the AI literature evolves rapidly. Important materials such as model cards, benchmark documentation, code repositories, technical reports, and updated checkpoints may fall outside conventional indexing, generating ascertainment bias toward peer-reviewed publications and highly visible preprints. In addition, consistent with the objectives of a scoping review, this study mapped concepts, methods, applications, and evidence patterns rather than estimating pooled model effects. Therefore, the review should not be interpreted as a meta-analysis of comparative model performance.

The methodological quality profile of the included studies also limits the strength of translational conclusions. The mean methodological quality score was 35.31%, with substantial dispersion, and only a minority of studies reached high-quality thresholds. This finding supports caution when interpreting broad claims about clinical utility, deployment readiness, or superiority of one method family over another. Heterogeneity in preprocessing, model families, benchmark datasets, split strategies, and evaluation metrics further complicates direct comparison. Studies varied in whether they used random, chromosome-held-out, site-held-out, or external validation designs; such differences can substantially affect estimated performance and generalizability. Reporting on interpretability, calibration, reproducibility, and governance was also uneven. Many studies mentioned interpretability tools, but fewer connected explanations to predefined biological or clinical criteria. Similarly, privacy, fairness, and protected health information were often acknowledged but less often operationalized through concrete governance procedures, federated designs, de-identification workflows, or auditing frameworks.

Reproducibility signals were also mixed. Code, model weights, dataset versions, preprocessing scripts, and curation procedures were not uniformly available. This issue is particularly important in foundation-model and LLM/RAG research, where model behavior may change with checkpoint versions, tokenizer updates, retrieval corpora, and prompt design. These limitations indicate constructive priorities for future research in the field. Future studies should use standardized and biologically meaningful benchmarks, report calibration and decision relevant outcomes in addition to discrimination metrics, adopt validation strategies that account for study site, technological platform, population ancestry and data source, and, whenever possible, make available versioned datasets, analytical code, model cards and evaluation protocols. For clinical and public health translation, prospective multicenter studies are particularly needed in areas such as variant interpretation, precision oncology, rare disease genomics, pharmacogenomics, infectious disease genomics and public health genomics. In the case of foundation models and large language model systems supported by retrieval augmented generation, future work should prioritize factual grounding, uncertainty quantification, transparency of retrieval sources, governance of protected data and oversight by human experts. These priorities arise directly from the main findings of this review, which show rapid growth, expanding methodological capacity, substantial heterogeneity, uneven validation and persistent gaps in interpretability, reporting and governance.

In summary, artificial intelligence in genomics has evolved from feature engineering and task specific models toward deep learning, multimodal systems, graph-based approaches and foundation model architectures. The field now demonstrates broad methodological diversity and promising performance in specific tasks. However, the evidence base remains heterogeneous and unevenly validated. Therefore, the most defensible conclusion is cautious: artificial intelligence is becoming increasingly central to genomic and multiomic research, but its responsible translation into clinical, research and public health contexts will depend on stronger external validation, clearer reporting, better calibration, biologically grounded interpretability, more inclusive datasets and explicit governance frameworks.

## 5 Final considerations

This scoping review mapped 1,040 studies on artificial intelligence in genomics and multiomics, providing a structured synthesis of method families, genomic modalities, application domains, evaluation practices, bibliometric trends, geographic distribution, topic-modelling patterns, and historical method trajectories. The findings show that AI–genomics research has expanded rapidly since 2017–2018 and now spans classical machine learning, deep learning, graph-based models, foundation models, and LLM/RAG-based systems. Rather than supporting a universal hierarchy of model superiority, the evidence indicates that model performance and suitability are task dependent, varying according to data modality, sample size, biological context, validation design, and reporting quality.

The main contribution of this review is to organize a highly heterogeneous and fast-moving field into interpretable evidence layers. Classical machine learning remains relevant for structured, feature-engineered, and interpretable genomic tasks; deep learning and graph-based approaches are prominent in sequence-to-function modeling, regulatory prediction, single-cell analysis, and multimodal integration; and foundation models and LLM/RAG systems are emerging in transfer learning, variant summarization, genomic question answering, clinical reporting, and retrieval-grounded knowledge synthesis. The review also highlights that AI–genomics research is geographically concentrated, methodologically uneven, and variable in evaluation practices, with recurrent gaps in external validation, calibration, interpretability, reproducibility, and governance.

Taken together, these findings position AI as an increasingly central component of genomic and multiomic research, but they also support a cautious interpretation of its translational readiness. The key take-home message is that the future value of AI in genomics will depend less on isolated performance gains and more on robust validation, transparent reporting, biologically meaningful benchmarks, interpretable outputs, inclusive datasets, and governance frameworks capable of supporting responsible clinical and public health use. By consolidating current applications, methodological patterns, and evidence gaps, this review provides a foundation for more reproducible, accountable, and clinically relevant AI-enabled genomics.

## Data Availability

The original contributions presented in this study are publicly available. All data underlying the analyses of methodological quality and results of the eligible studies included in this scoping review are deposited in the Open Science Framework (OSF) and can be accessed at: https://osf.io/uexzh. The full search strategies used for each database are provided in Supplementary File 1: Search Strategies, which accompanies this article as Supplementary Material.
