# Supplementary material for: Artificial intelligence for genomic science: a scoping review of concepts, architectures, applications, and open challenges
Source: Front Bioinform. 2026 Jul 3;6:1829576. doi: 10.3389/fbinf.2026.1829576 (PMC13375738; doi:10.3389/fbinf.2026.1829576)
Supplement: Supplementary file 1 [file Supplementaryfile1.docx]

Supplementary Material

# Supplementary Data

Supplementary File 1: Search Strategies:

Medline/pubmed = (((((((Genomics[Title/Abstract]) OR (Comparative Genomics[Title/Abstract])) OR (Genomics, Comparative[Title/Abstract])) OR (Structural Genomics[Title/Abstract])) OR (Genomics, Structural[Title/Abstract])) OR (Functional Genomics[Title/Abstract])) OR (Genomics, Functional[Title/Abstract])) AND ((((((((((((((((((((Artificial Intelligence[Title/Abstract]) OR (Intelligence, Artificial[Title/Abstract])) OR (Computer Reasoning[Title/Abstract])) OR (Reasoning, Computer[Title/Abstract])) OR (AI (Artificial Intelligence[Title/Abstract]))) OR (Machine Intelligence[Title/Abstract])) OR (Intelligence, Machine[Title/Abstract])) OR (Computational Intelligence[Title/Abstract])) OR (Intelligence, Computational[Title/Abstract])) OR (Computer Vision Systems[Title/Abstract])) OR (Computer Vision System[Title/Abstract])) OR (System, Computer Vision[Title/Abstract])) OR (Systems, Computer Vision[Title/Abstract])) OR (Vision System, Computer[Title/Abstract])) OR (Vision Systems, Computer[Title/Abstract])) OR (Knowledge Acquisition (Computer[Title/Abstract]))) OR (Acquisition, Knowledge (Computer[Title/Abstract]))) OR (Knowledge Representation (Computer[Title/Abstract]))) OR (Knowledge Representations (Computer[Title/Abstract]))) OR (Representation, Knowledge (Computer[Title/Abstract])))

Embase: ( 'genomics'/exp OR 'comparative genomics'/exp OR 'structural genomics'/exp OR 'functional genomics'/exp OR (genomics OR "comparative genomics" OR "structural genomics" OR "functional genomics"):ti,ab ) AND ( 'artificial intelligence'/exp OR 'machine learning'/exp OR 'deep learning'/exp OR 'neural network'/exp OR 'computer vision'/exp OR 'knowledge acquisition'/exp OR 'knowledge representation'/exp OR ("artificial intelligence" OR "machine intelligence" OR "computational intelligence" OR "computer reasoning" OR "reasoning, computer" OR "computer vision system*"OR "vision system*, computer" OR "knowledge acquisition" OR "knowledge representation" OR "large language model*" OR "foundation model*" OR transformer*):ti,ab ).
